# Effects of Preheating on Internal Modification and Welding Strength of Glass by Ultrafast Laser Pulses

**DOI:** 10.3390/mi17050507

**Published:** 2026-04-22

**Authors:** Rafid Hussein, Shuting Lei

**Affiliations:** Department of Industrial and Manufacturing System Engineering, Kansas State University, Manhattan, KS 66506, USA; rafidh@ksu.edu

**Keywords:** self-focusing, transmission laser welding, plasma-modified, heat-affected zone

## Abstract

Glass preheating prior to laser scanning is expected to enhance internal modification morphology; however, its effect on weld seam topology and welding strength have not been investigated. In the current work, the effects of preheating on ultrafast laser (184 fs and 10 ps) internal modification and welding strength of borosilicate glass slides are investigated. For the internal modification experiments, pulse energy of 30–100 µJ and repetition rate of 10 kHz are used by focusing a laser beam at the interface of optically contacted slides at room temperature (RT ≈ 23 °C), 150 and 200 °C. Welding is conducted by a pulse energy of 4.5–18 µJ and repetition rate of 200 kHz using pre-clamped glass slides with a scanning speed of 10 mm/s at RT and 150 °C. Also, for welding, the optimum number of scans and hatching spacing are identified. Filamentation experiments show that discoloration is not significant when preheat temperature reaches 200 °C. Compared to 10 ps, pulse duration of 184 fs can produce a 19% narrower plasma-modified region at both RT and 150 °C and a 13% wider heat-affected zone at 150 °C. Welding using optimum conditions of 5 scans and 200 µm hatch, and “crack-free” laser parameters produces an average strength of: 50 ± 3.2 MPa at RT and 40 ± 2 MPa at 150 °C for 184 fs compared to 35 MPa at RT and 32 MPa at 150 °C for 10 ps, using 10 replicates each. However, the welding strength upon preheating to 150 °C using 184 fs is still 25% higher compared to average reported laser welding bonding strength, while the 10 ps strength is within the reported average. The enhanced welding strength for 184 fs can be attributed to reduced microcracking, especially when “crack free” combinations are utilized.

## 1. Introduction

Transmission laser glass welding utilizes localized melting and gap bridging taking place at the interface of contacted transparent surfaces by generating a tear-drop shaped modification [1]. The tear-drop bridges the interfacial gap, producing a permanent weld, mainly due to a plasma-modified core of the tear-drop [2]. Nonlinear absorption triggers the initiation of the tear-drop at the focal position. The tear-drop grows towards the incident laser by avalanche ionization and plasma absorption. The incident intensity at the focal position should exceed the intensity threshold for nonlinear absorption initiation [3].

Optimization of the incident beam parameters is a critical step for producing defect-free (crack-free) weld seams with enhanced tear-drop morphology and strength [4]. Investigations of tear-drop morphology show that the tear-drop’s height and width are increased when the pulse repetition rate is increased in the range of 50 to 1000 kHz. By increasing the pulse energy in the range of 1 to 11 µJ and the repetition rate, nonlinear absorptivity is also enhanced from 40 to 95% [5]. Decreasing scanning speed can also increase the tear-drop width and height, which is significant when the speed is decreased by orders of magnitude for higher pulse energy levels [6]. Absorption volume simulations of a 20 µJ laser beam using a numerical aperture NA of 0.4 to 1.2 revealed that a constant-width tear-drop can be achieved with nonlinearly decreasing height and an aspect ratio of height-to-width, where the aspect ratio can reach 1 for NA = 1.2 [7]. The effect of NA on the tear-drop morphology can be even more complicated if the spherical aberration and self-focusing are considered, especially when the focal position is located deeper inside thick glasses [8].

In the authors’ previous papers [9,10], it has been determined experimentally that preheating glass to 150 and 200 °C prior to laser scanning can increase tear-drop height and width. In the current work, the authors first introduce an experimental investigation of the preheating temperature effects on laser absorptivity under self-focusing modification. The width of the tear-drop’s heat-affected and plasma-modified zone are then investigated at RT and 150 °C for pulse energy of 4.5–18 µJ and duration of 184 fs and 10 ps.

Welding defects (cracks) formation can also depend on the initial gap before laser scanning; optical contact with a gap < 200 nm can produce crack-free weld seams as tensile stresses generated by the glass melt are balanced up on solidification [1]. Pre-welding gap between glass substrates can be controlled using mechanical clamping to get partial optical contact where Van der Waals forces temporarily join the contacted surfaces [11]. Full optical contact is also achievable by high-quality polishing, but it is technically expensive [12].

A number of parallel scans are recommended to weld glass-to-glass to produce consistent strength measurements [13] and minimize the uncertainty effects stemming from variations in sample surface quality and thickness. Jia et al. [14] investigated welding of soda lime glass and reported a tensile shear strength of 3.3 and 6.3 MPa using 10 and 15 welding scans, respectively, while Chen et al. [2] used 40 scans and reported a 1325 N compression shear force for borosilicate glass. Richter et al. [15] did not report the number of welding scans in their experiments, which corresponded to 72.9 and 39 MPa for welding of fused silica and borosilicate glass, respectively. Zhang et al. [16] utilized a line-to-line (hatch) spacing of 250 µm, producing a shear strength of 10.9 MPa; however, when the spacing was reduced to 120 µm, the strength increased to 17.4 MPa. Tian et al. [17] and Chen et al. [18] used a scan spacing of 50 and 200 µm producing a shear force and strength of 76 N and 18 MPa, respectively. Most recently, Jiang et al. [19] conducted a quasi-synchronous welding using 5 scans of 10 µm hatch, producing a weld compression lap-shear strength of 21.42 and 16.33 MPa for a pre-welding gap of 1 and 5 μm, respectively.

Effects of preheating temperature on the welding quality and strength have not yet been investigated in the literature. Additionally, hatch spacing effects and its correlation to laser-induced cracking defects have not been reported so far.

In the current work, preheating temperature effects on filamentation modification pattern, absorption rate, and welding defects and strength are investigated. Room temperature, 150 and 200 °C are denoted as RT, MT, and HT, respectively. Absorptivity rate is calculated using incident and transmitted pulse energy measurements in the z-direction after the beam exits contacted glass slides at three distances: *dl* = 103.5, *dp* = 292, and *dh* = 502 mm. Welding experiments are performed at RT and 150 °C using an optimum hatch distance *h* and number of scans with 10 replicates for each parameter combination where welding defects are analyzed and the corresponding strengths are determined using a single-lap compression shear test.

## 2. Absorption Rate Determination and Modification Pattern

**Figure 1:**
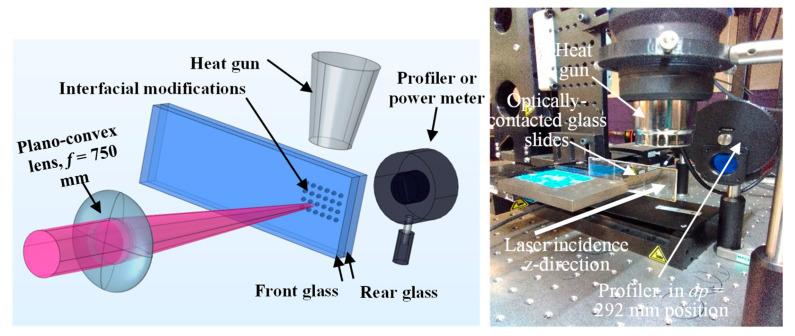
Experimental setup for transmitted pulse energy *E_t_* measurements at *dl* = 103.5, *dp* = 292, and *dh* = 502 mm; the setup shows measurement of *E_t_* at *dp* = 292 mm.

In the current work, absorption rate measurement is performed by focusing a laser beam at the interface of optically contacted glass workpieces and by measuring the transmitted laser power for a known incident laser power, as shown in Figure 1. Absorption rate measurement techniques assume negligible reflection and scattering due to plasma generation [20]. Absorption rate *A* can be calculated using Equation (1) [21]:

(1) $$A=\frac{E_{a}}{E_{i}}=\frac{E_{i}{\left(1-R\right)}^{2}-E_{t}}{E_{i}\;(1-R)}\;$$

where *E_a_*, *E_i_*, *E_t_* and *R* are the absorbed, incident, transmitted pulse energy, and reflectivity of the glass surface, respectively. In the literature, determination of the absorption rate using this approach has been implemented for laser intensities exceeding the threshold [5,20] where tear-drops are generated, which are technically permanent damages. Under this damage condition, the measured *E_t_* when the tear-drop modification happens could affect the calculated absorption. Heat accumulation can also affect the measurements when pulse repetition rate exceeds 100 kHz. On the other hand, absorptivity can also be measured under conditions of self-focusing as nonlinear propagation inside the glass is still happening, which is a general procedure for Z-scan tests [22]. However, for Z-scan self-focusing conditions, the sample’s thickness should be smaller compared to the lens’ Rayleigh range [23] and the repetition rate should be in the neighborhood of 10 kHz to minimize accumulation heating [24]. Experiments of this section are conducted under self-focusing condition where laser peak power exceeds the critical peak power of the glass but not the tear-drop formation, as discussed below and elaborated in Section 4.1.

For the calculations of *A* and glass modification patterns, a laser system of wavelength λ = 1028 nm producing a beam spot size *D* = 5 mm is utilized. The collimated laser beam is focused using a plano-convex lens of focal length *f* = 750 mm at the interface of two optically contacted glass slides of 1 mm thickness each, as shown in Figure 1. First, the beam spot size *w*_0_ at the focal plane is measured in air using a camera profiler. The measured spot size is *w*_0_ = 112.71 µm, which is only 2.71 µm higher compared to the calculated spot size of *w*_0_ = 2M^2^λ*f*/π*D* for a Gaussian beam, where M^2^ = 1.1. An incident laser beam of 0.3–1 W (30–100 µJ), 184 fs, and 10 kHz for power (pulse energy), pulse duration, and repetition rate, respectively, is used to run the absorption rate experiments at RT and HT. Two optically contacted glass slides are edge-sealed using a commercial glue, then a heat gun is utilized to preheat the slides to 200 °C, which is measured using a thermal camera. Prior to laser scanning, preheating by a heat gun can trigger a background effect on the power meter measurements. These effects are accounted for by subtracting the background measurements from the real measurements after laser exposure. The transmitted power is measured after the beam exits the glass at three levels of z-distance: 103.5, 292, and 502 mm; these are measured from the outer surface of the rear glass slide to the meter, (Figure 1). Three replicates for RT and the other three for HT are utilized. For each replicate, when a distance level or a pulse energy is reset, a new intact spot of the glass replicate is utilized to create the required modification and acquire the measurements.

Commercial borosilicate glass slides of dimensions 76 × 25 × 1 mm^3^ under optical contact are utilized for experimental investigations of modification patterns and laser welding. For welding experiments, the glass slides are cut to 38 × 25 × 1 mm^3^. Material properties are listed in Table 1 below.

## 3. Glass Welding and Single-Lap Compression Shear Test

**Figure 2:**
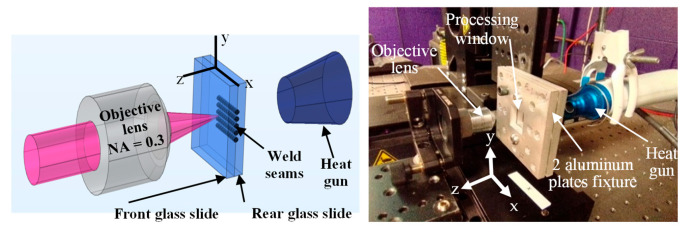
Experimental setup of glass welding at RT and MT; for MT the gun heats up the glass slides from rear side which is attached to the stage that scans in x-direction.

Welding experiments are performed to weld two glass slides placed between two aluminum plates as shown in Figure 2. Two bolts are used to secure the glass slides at a processing window of 23 × 23 mm^2^ where the bolts are hand-tightened prior to laser scanning. Pre-weld optical contact regions are observed but are limited and varied in location inside the window due to sample-to-sample variations. First, the distance between the lens and glass slides is optimized by initial welding experiments to locate the focal position at the welding condition. Then, welding experiments are conducted at RT and MT using the combinations of pulse energy, pulse duration, scanning speed, and hatch distance listed in Table 2.

The glass temperature upon preheating is monitored by a thermal camera to measure rear and front aluminum and glass surfaces temperature and ensure that it does not build up excessively. First laser scan starts when the maximum temperature of the rear glass reaches around 140 °C, while the maximum temperature may rise to 155 °C upon finishing the last welding scan. More details are included with FEA simulations in a previous work [10]. Similar procedure is followed for the internal modification preheating. There have been technical limitations over the pulse energy and preheat temperature levels for a successful welding. For the MT conditions, the 14 and 18 µJ pulse energy produce significant cracking during welding. Similar cracking during welding is also noticed for almost all pulse energy levels for HT conditions. Accordingly, these conditions are excluded.

The selection of *h* for the 184 fs and 10 ps is optimized to minimize the possibility of crack extension perpendicular to parallel scans for the welded samples to make a fair comparison between fs and ps conditions. Initial welding experiments show that *h* should be set proportional to pulse energy if the cracks perpendicular to the parallel laser scans should not connect to each other. If these cracks connect, then strength is reduced drastically, which is confirmed by initial strength measurements.

**Figure 3:**
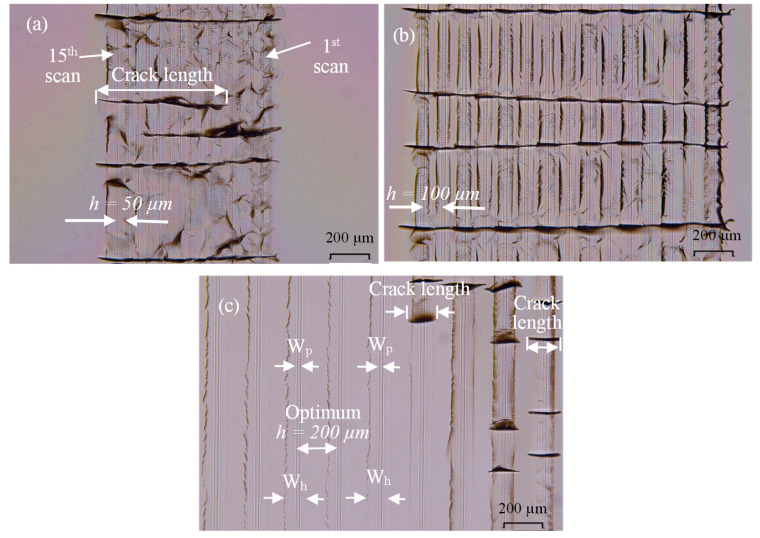
Effects of hatch *h* on crack connection of three 15-scan welded samples at RT: (**a**) *h* = 50 µm, (**b**) *h* = 100 µm, and (**c**) *h* = 200 µm. Measurements of *W_p_*, *W_h_*, and microcracks length are also shown.

Figure 3 shows microscopy images of the interface of samples welded by 10 µJ, 10 ps, 200 kHz, 10 mm/s and 15 scans with *h* of 50, 100, and 200 µm as marked by a, b, and c, respectively. An interesting observation is that cracks produced by the initial scans (1st scan and so on) have extended perpendicular to the weld seams when *h* < 200 µm, which is later selected as the optimum hatch distance. Figure 3c shows the effect of using *h* = 200 µm where cracks do not extend across weld seams in this case, which is also confirmed by four other replicates.

**Figure 4:**
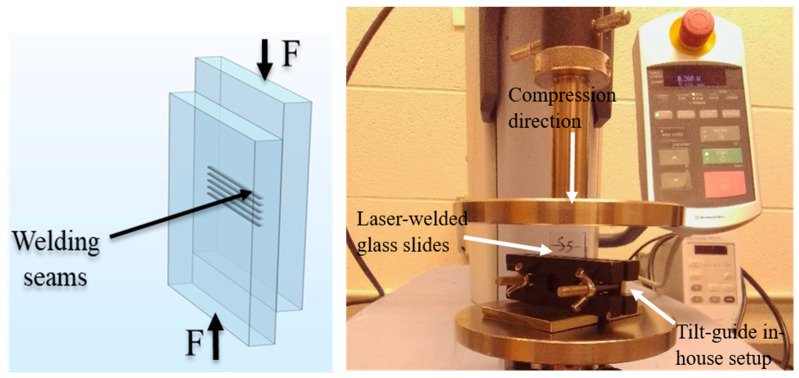
Experimental setup of lap shear strength testing; a tilt guide is utilized to avoid sample tilt during compression loading.

A Shimadzu universal testing machine of 5 kN maximum load is used to conduct single-lap compression tests to determine the lap shear strength of the welded slides using an in-house made fixture, as shown in Figure 4. A platen speed of 0.02 mm/s is used and the test is stopped after the weld seam failure when a catastrophic drop in shear force happens. Initial samples are welded by 5 and 15 scans; however, testing of the 15-scan welded samples shows that failure occurs at the bulk glass, not the welded interface. Apparently, the weld seams are strong enough causing bulk glass slides welded with 15 scans to break prior to shearing of the weld seams. Accordingly, the 15-scan welding scheme is not considered for further investigation. Figure 3c also shows the determination of width of the plasma-modified *W_p_* and the heat-affected *W_h_* zone, which will be discussed in Section 4.3.

## 4. Results and Discussion

### 4.1. Glass Internal Modification Pattern

**Figure 5:**
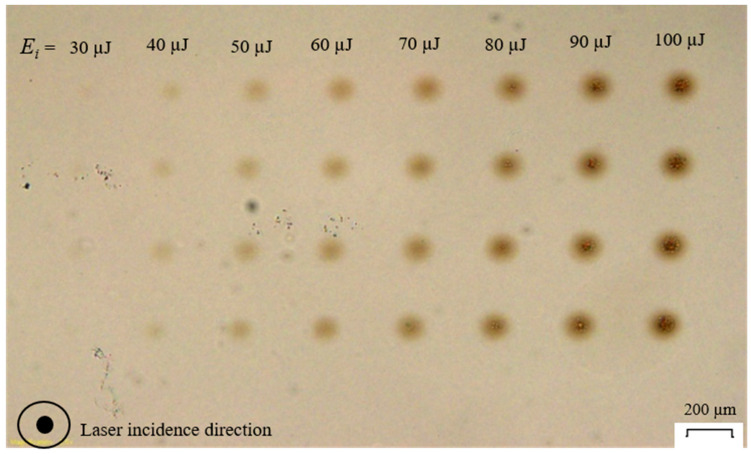
An interface of a glass slide under optical microscope after laser transmission at RT showing four modification sets per pulse energy for consistency.

**Figure 6:**
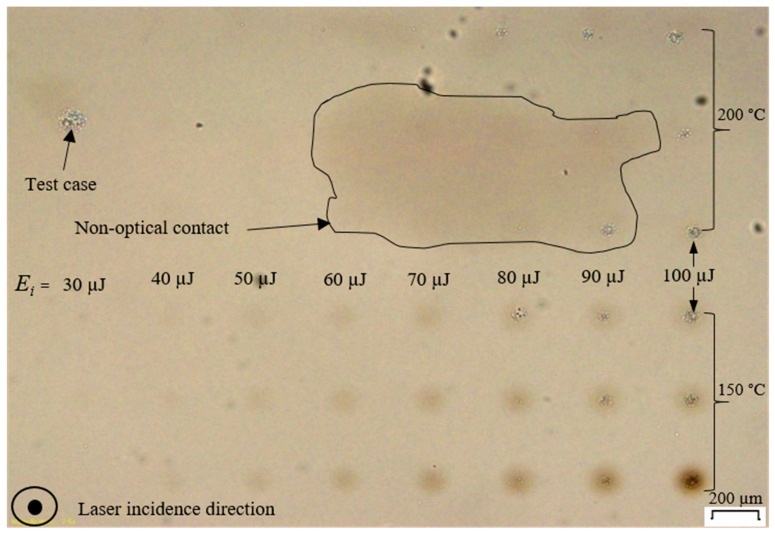
An interface of a glass slide after laser transmission at MT and HT showing three modification sets per pulse energy per temperature for consistency.

Figure 5 and Figure 6 show modifications at the interface of a front glass sample of the contacted pair created at RT, MT, and HT. For the RT case, apparent circular discoloration happens under the effects of laser-glass interaction, the intensity of which being proportional to the pulse energy. This discoloration is attributed to the color-center formation where a change in refractive index of borosilicate glass happens due to self-focusing [26]. Additionally, a clustered-particulate modification centered at the discoloration circles is also observable, especially for the 80–100 µJ pulse energy. For the MT and HT, the discoloration is not significant for the former and not discernable for the latter. However, the clustered-particulate modification is still remarkable.

**Figure 7:**
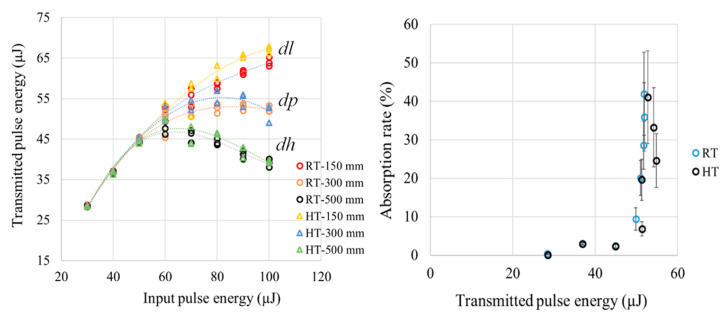
Transmitted pulse energy measurements of three replicates using three positions of power meter at both RT and HT, and the calculated absorption rate %.

The analysis below explains the effects of pulse energy on discoloration intensity. The incident laser beam of *E_i_* = 30 µJ has a peak power of around 163 MW, which is around 50 times the critical peak power for self-focusing of 3.2 MW for borosilicate glass, as reported in [3]. The peak power can be calculated by P_peak_ = *E_i_/t_p_*; however, glass can only absorb a portion of the incident pulse energy, which is estimated by the absorption rate analysis of Section 4.2. From Figure 7 (Section 4.2), *A* ≈ 1% for *E_i_* = 30 µJ; P_peak_ ≈ 1.6 MW, which is lower than the critical peak power for self-focusing. This explains the absence of discoloration for the 30 µJ at RT in Figure 5. For *E_i_* > 30 µJ, Figure 7 shows that it is possible to exceed the critical peak power based on the absorption rate level; for example, when *E_i_* = 40 µJ, *A* ≈ 3% and P_peak_ ≈ 4.8 MW, which exceeds the critical peak power. In all cases of Figure 5 and Figure 6, the incident beam intensity *I* does not exceed the intensity threshold of 2.15 × 10^13^ W/cm^2^ [7] for borosilicate glass. Intensity *I* is calculated by *I* = *F/t_p_* where *F = E_i_/A_s_* is the incident laser beam fluence and *A_s_* is the spot area.

Apparently for RT, MT, and HT, the discoloration has a higher intensity at the center of the modification circles, which abates as the radial distance from the circles’ center increases. Assuming that the filamentation modification of glass still has a Gaussian or a semi-Gaussian intensity, the radial dependence of discoloration intensity can be correlated to the intensity’s radial distribution.

The discoloration generation is attributed to the formation of color centers due to trapped electrons/holes under the exposure of femtosecond pulses [26,27]. These electrons/holes are unstable which can recombine upon annealing heating.

Free electrons in dielectric materials like glass are generated as a result of laser material interaction. When an incident laser beam of wavelength λ is transmitted through a glass sample, laser energy is absorbed by valence electrons of the dielectric material. If the photonic energy is high enough to move electrons from the valence band to the conduction band, a multiphoton (N-photon) ionization phenomenon takes place and generates free electrons. The photon energy E_photon_ is inversely proportional to λ, and the number of photons, N required to trigger one electron can be determined by N = E_gap_/E_photon_, where E_gap_ is the band gap of the dielectric material. The freed electrons by multiphoton absorption can also collide to other valence electrons moving them from the valence band to the conduction band and generating more free electrons by avalanche ionization [19].

The observations in Figure 5 and Figure 6 suggest two possible scenarios: either concurrent discoloration formation and recombination happens or color center formation does not take place upon preheating. In the current work, preheating above RT where the temperature difference of 125 for MT and 175 °C for HT can still relax the self-focusing-assisted discoloration but not to the level that stops self-focusing, which is still significant in terms of clustered-particulate interfacial modifications. Figure 6 has a feature marked as a “Test case”, which is used as a reference for the HT modification’s location when inspected under microscopy. Also in Figure 6, the “Non-optical contact” contour marks a region at the interface where optical contact is relaxed/lost under the effect of preheating during laser scan, which did not affect the outcomes of the modification pattern creation and analysis.

### 4.2. Absorption Rate Measurements

Figure 7 shows the measured transmitted pulse energy *E_t_* and the calculated absorption rate *A*% for the incident pulse energy *E_i_* in the range of 30–100 µJ at three far-field positions in z-direction after the beam exits the glass slides: *dl* = 103.5, *dp* = 292, and *dh* = 502 mm. In Figure 7 (right), *A*% is calculated for each *E_i_* using Equation (1) by taking *E_t_* as the average of nine measurements, three replicates at all three far-field positions at RT. Similarly, there are nine other measurements for HT at all three far-field positions. From Figure 7(left), the *E_t_* can be linearly correlated to *E_i_* at RT and HT up to 50 µJ while the absorption rate is not significantly affected by glass temperature within this pulse energy range as shown in Figure 7 (right); *A* is in the range of 1–3%. At HT and for *E_i_* > 50 µJ, the *E_t_* is around 5% higher for all positions compared to the RT up to the highest *E_i_* of 100 µJ. Assuming that the level of clustered-particulate damage is not affected by the temperature as shown in Figure 5 and Figure 6, the lower level of *E_t_* in the RT curve for *E_i_* > 50 µJ (Figure 7, left) can be attributed to the discoloration. The discoloration at RT contributes to higher absorbed energy *E_a_* for *E_i_* > 50 µJ compared to the HT, which is confirmed by the marginal increase in *A* % as shown in Figure 7 (right).

For RT and HT, as the distance is increased from *dl* to *dh*, the measured *E_t_* decreases significantly for pulse energy of Ei > 50 µJ (Figure 7, left). Additionally, the reduction in *E_t_* when the distance is increased is proportional to pulse energy. This distance-dependent reduction in the measured *E_t_* (Figure 7, left) is attributed to the scattering effect of the clustered-particulate modification formation at RT and HT, which is proportional to pulse energy and does not change with temperature, as shown in Figure 5 and Figure 6.

### 4.3. Weld Seam Topology: Heat-Affected and Plasma-Modified Region

**Figure 8:**
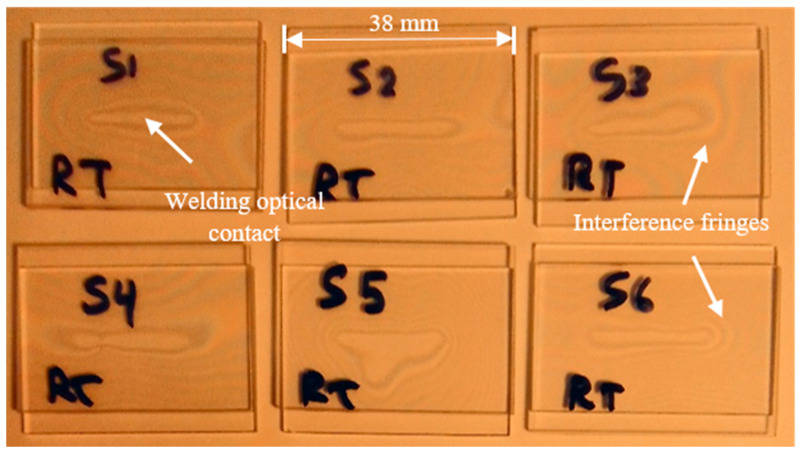
Six welded pairs of glass slides using 10 µJ and 10 ps at RT showing the welding optical contact of 5 scans per sample and the surrounding interference fringes.

Welding of glass slides is performed utilizing the parameter combinations of Table 2. Ten replicates of 38 × 25 × 1 mm^3^ are welded for each treatment combination using five parallel scans, repetition rate of 200 kHz, and scanning speed of 10 mm/s at RT and MT utilizing the setup of Figure 2. Then, the replicates are inspected by optical microscopy to capture images of the interfacial weld seam. Figure 8 shows six welded glass slides having optical contact created at the weld seams surrounded by interference fringes.

After welding, the width of the heat-affected zone, *W_h_*, the width of the plasma-modified region, *W_p_*, and the length of cracks are measured using imageJ (1.54s). Figure 3a,c show sample measurements of *W_h_*, *W_p_*, and crack length. Cracks are projected where only straight measurements are taken, even for tilted cracks, to avoid the complexity of measuring branched, oblique cracks, and cracks with different opening directions. Only cracks that are perpendicular to the weld seam direction are considered for the analysis in Section 4.5 as these cracks represent the most significant encountered defect type for the inspected samples that affects the strength.

**Figure 9:**
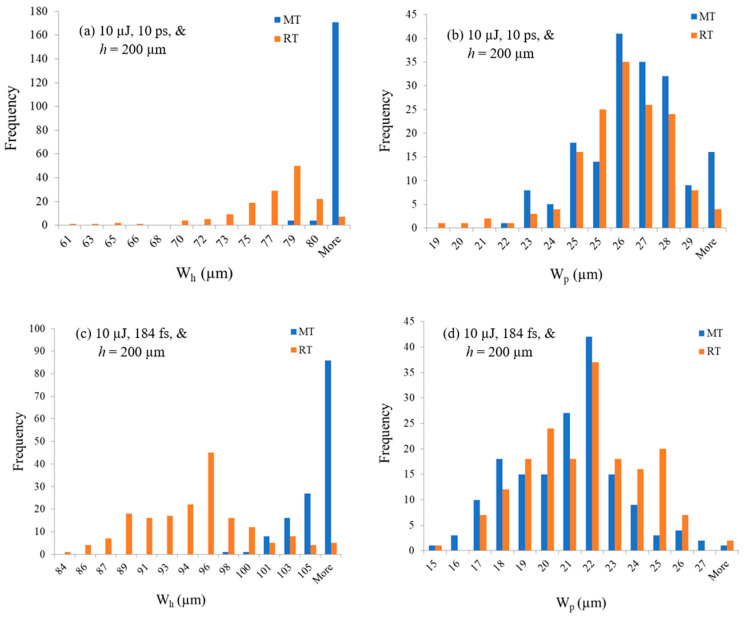
Histograms of measured *W_p_* and *W_h_* for 184 fs and 10 ps at RT and MT, measurements of 10 replicates per histogram per temperature.

Figure 9 shows the measurements of four groups of ten replicates. The *W_h_* and *W_p_* measurements at RT of Figure 9a,b are for the same group of ten replicates. Another group of ten replicates is utilized to measure *W_h_* and *W_p_* at MT of Figure 9a,b. Similar measurements are made for the pulse duration of 184 fs and shown in Figure 9c,d. A minimum of three measurements are acquired for each scan of the five weld seams per sample; meaning that at least 15 measurements are taken per sample. Qualitatively in Figure 9a,c, the tear-drop width, which is equal to *W_h_* is dependent on the preheating temperature; the MT measurements are shifted within the top 50% of the range compared to the RT condition. In Figure 9a,c and at MT, *W_h_* > 78.69 µm for the 10 ps while *W_h_* > 97.81 µm for the 184 fs. Comparatively, there is an insignificant dependence of *W_p_* on preheating temperature for both 10 ps and 184 fs welding conditions, as shown in Figure 9b,d.

**Figure 10:**
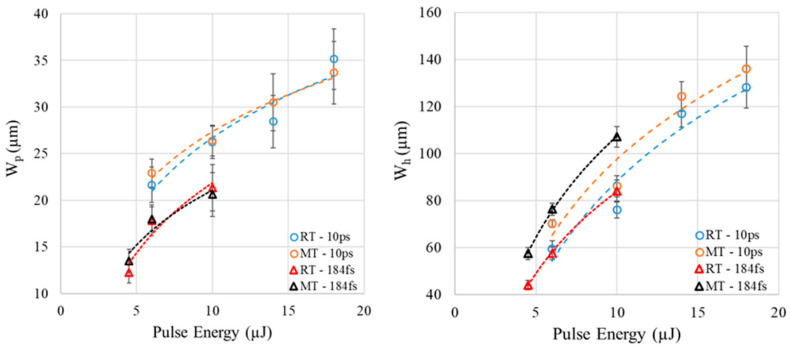
Average measurements and deviation of *W_p_* and *W_h_* for Figure 9 histograms.

To better understand the dependence of *W_h_* and *W_p_* on preheating temperature and pulse duration or incident intensity, average measurements of *W_h_* and *W_p_* for the pulse energy of 4.5, 6, 10 µJ and 6, 10, 14, 18 µJ for the femtosecond and picosecond pulse durations, respectively, are plotted in Figure 10.

Interestingly, there are two important outcomes from Figure 10: first, the 10 ps plasma-modified seams where glass welding is expected to occur have shown a wider *W_p_* compared to the 184 fs; *W_p_* is around 19% higher for the 10 ps compared to 184 fs within the range of 6–10 µJ at both temperatures. Second, *W_h_* is not dependent on pulse duration at RT while it depends on temperature at MT; at MT, *W_h_* is around 13% higher for the 184 fs compared to the 10 ps within the range of 6–10 µJ. In short, *W_p_* depends on pulse duration and not temperature, while *W_h_* depends on temperature and pulse duration for any preheating temperature higher than RT.

**Figure 11:**
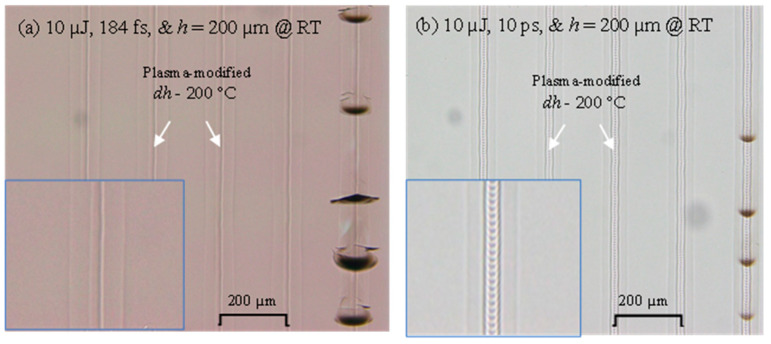
A comparison of plasma-modified regions of 184 fs and 10 ps weld seams showing a stair-case topology for 10 ps, which happens for all pulse energy at RT and MT.

**Figure 12:**
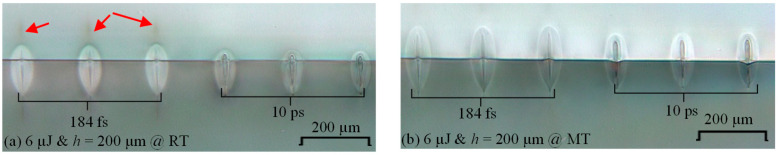
Effects of temperature on discoloration and plasma-modified morphology explaining a possible reason for the stair-case topology of 10 ps weld seams at RT and MT.

Another finding for the comparison of weld seams generated by 184 fs and 10 ps pulse durations can be seen in Figure 11. The plasma-modified region where welding is expected to occur for the 10 ps has a clear pulse-to-pulse staircase topology compared to the 184 fs where the staircase topology is not discernable, as shown in the magnified inserts. This effect can be seen for all replicates of RT and MT cases; apparently it does not depend on temperature. The staircase is not visible for the 184 fs due to the fact that the top portion of the plasma-modified region has a pin-shaped topology compared to a curve-shaped top portion for the 10 ps weld seams, as shown in Figure 12. Additionally, the red arrows point to the discoloration due to self-focusing at RT and 184 fs, which does not happen at MT and 184 fs, and both temperatures at 10 ps.

### 4.4. Single-Lap Compression Shear Strength

**Figure 13:**
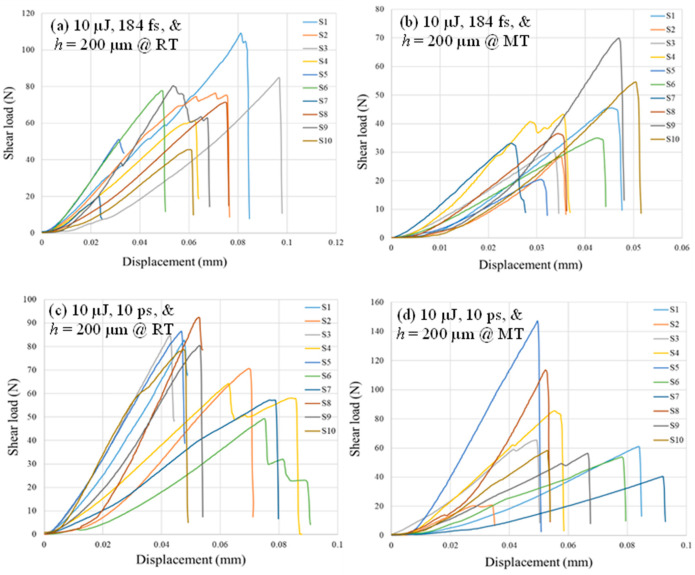
Sample load–displacement curves of single-lap shear tests showing fitted elastic and post-failure portions of the curve.

Figure 13 shows load–displacement curves of the lap shear strength testing for samples welded with 10 µJ pulse energy, 5 scans, and *h* = 200 µm at RT and MT. Figure 13a–d are for the 184 fs and 10 ps pulse duration, respectively. Variations between samples produced scatter in the load–displacement behavior. Effects of pre-weld and post-weld partial optical contact take place for the elastic portion prior to maximum load in terms of a limited zigzag elastic behavior. Post maximum load, there are progressive failures for some samples because breaking of weld seams introduces glass flakes at the interface between samples and could increase local friction forces while under the compaction of the tilt guide. However, since the major outcome of the single-lap shear test is the maximum shear load, the progressive failure should not have a significant effect.

**Figure 14:**
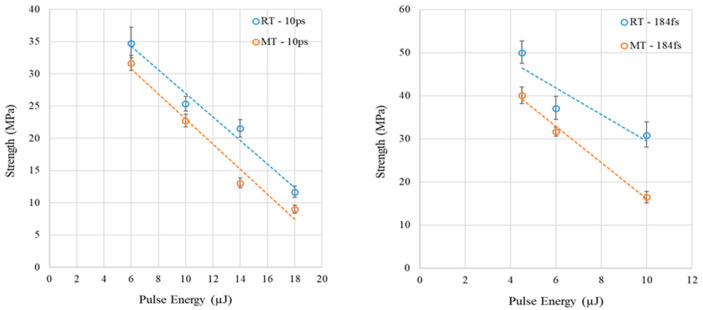
Average shear strength and deviation of weld seams vs. pulse energy for 184 fs and 10 ps at RT and MT; strength decreases with pulse energy and preheating.

Shear strength is calculated by dividing the maximum load by the shear area. The shear area is determined by the product of *W_p_* and the seam length of five scans, which is around 23 mm per scan. *W_p_* is determined by the measurements of Section 4.3; *W_p_* is taken as the average of 15 measurements per sample for strength calculations. To exclude strengths that are abnormally larger or smaller compared to the measurements’ bounds, the 1st and 3rd quartile outlier analysis is performed for the calculated shear strengths. Filtered strengths data are depicted in Figure 14 for the 184 fs and 10 ps at RT and MT.

Surprisingly, the shear strength of samples welded at RT is higher than the shear strength for samples welded at MT regardless of the incident beam pulse duration, as shown in Figure 14. However, when a linear trend is fitted to the strength vs. pulse energy, an enclosed region is formed between the RT and MT lines, which is wider for the 184 fs. The fitted lines show that the strength at RT is between 11 and 66% higher than that at MT for 6–18 µJ using 10 ps pulse duration. Similarly, the strength at RT is between 19 and 83% higher than that at MT for 4.5–10 µJ using 184 fs pulse duration. Apparently, the effect of preheating temperature on welding strength reduction is more significant at 184 fs compared to 10 ps. To explain this finding, cracks generated due to laser glass welding are analyzed for all replicates of 184 fs and 10 ps pulses.

The difference in strength of the “crack-free” combinations (see Section 4.5) of Figure 14 shows that the 184 fs pulse duration exhibits a maximum strength of around 50 MPa compared to 35 MPa of 10 ps. This difference can be caused by laser-induced defects at smaller scales where the connectivity of the ring structures of glass material decreased at specific locations of nonbridging oxygen hall centers and compacted at other locations, which can impede the propagation of cracks generated after mechanical loading application [7]. This scenario can also explain a possible reduction in fracture toughness that can happen for laser glass welding implemented at MT using “crack-free” combinations where preheating contributes to microcrack initiations and lower fracture toughness. The possible reduction in fracture toughness is higher for the 10 ps compared to the 184 fs; however, this hypothesis for “crack-free” and “crack-rich” conditions is yet to be proved in future work.

Welding of preheated substrates using “crack-free” process parameters producing promising strengths for the 184 fs and 10 ps, which are 25% and 10% higher compared to average laser welding bonding strength of 32 MPa reported in [18]; the 32 MPa strength is the average of nine different investigations.

### 4.5. Post-Welding Crack Measurements

**Figure 15:**
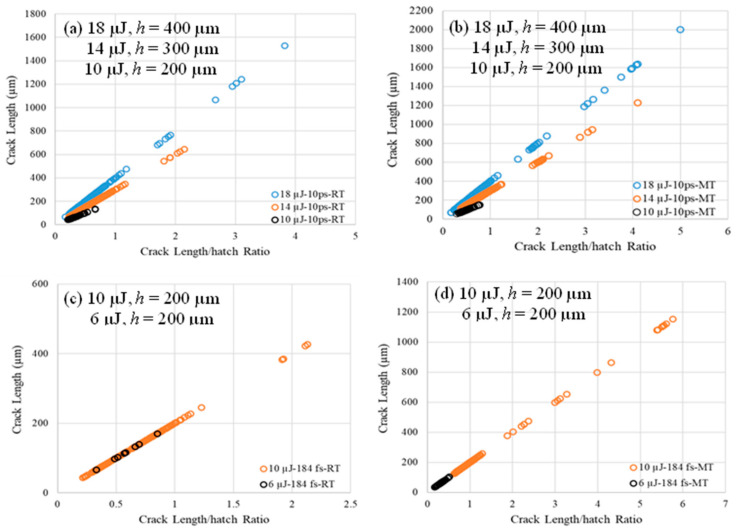
Post-welding crack measurements for the combinations of Table 2, 4.5 µJ at 184 fs and 10 µJ at 10 ps (not plotted) have shown less than 10 small microcracks for only 3 replicates out of 10 and hence are considered as “crack-free” combinations.

Figure 15 shows the measurements of projected perpendicular cracks for 6 and 10 µJ of 184 fs and 10, 14, and 18 µJ of 10 ps at RT and MT, respectively. Pulse energy of 4.5 µJ at 184 fs and 6 µJ at 10 ps are denoted as “crack-free” combinations that have not shown significant cracking; number of cracks is around 10 for no more than two replicates. This is why these cracks are not reported in Figure 15; however, Figure 14 still shows a higher strength for RT compared to MT for 4.5 µJ at 184 fs and 6 µJ at 10 ps.

In Figure 15, crack length measurements are plotted against crack length-to-hatch ratio. The number of cracks, their lengths relative to hatch, and the slope, which represents the hatch can be investigated. At both RT and MT, when a group of combinations of 6 µJ for 184 fs and 10 µJ for 10 ps are used to weld glass slides, significant cracks are generated; however, their lengths are still not exceeding the respective hatch; crack length-to-hatch ratio does not exceed 1, as shown in the dark colored circles of Figure 15a–d. Among this group’s four combinations, the 6 µJ for 184 fs at RT shows an average strength of around 42 ± 1 MPa (based on trend line of Figure 14) compared to 27 ± 1.1 MPa for 10 µJ for 10 ps (based on trend line of Figure 14). Comparing these two cases, the 184 fs one has a 55% higher strength compared to the 10 ps. In Figure 15, comparing the MT cases to their counterparts of RT cases, MT welding produced more and longer cracks compared to RT conditions, which could be the reason behind lower strengths for welding at MT shown in Figure 14, assuming that fracture toughness does not change significantly by temperature.

## 5. Conclusions

The role of preheating in laser glass welding under ultrafast laser pulses of 10 ps and 184 fs is investigated in the current work. While the pulse energy range for welding of 4.5–18 µJ at 184 fs produces self-focusing discoloration, similar pulse energy range at 10 ps does not, as the incident peak power does not exceed 3.2 MW for 10 ps. Investigating the effects of preheating on absorption rate when only self-focusing is significant can still explain the effect of preheating on nonlinear absorption, without the generation of tear-drops. Based on this hypothesis, outcomes of modification pattern experiments and absorption rate determination using 184 fs are applicable for preheated glass welding under 10 ps and 184 fs pulse durations. Internal modification analyses show that preheating has a significant effect on inhibiting discoloration due to color center formation associated with self-focusing filamentation; however, preheating within the current range does not seem to enhance absorption rate. The particulate-clustered damages that are significant at pulse energies higher than 50 µJ may not happen during welding, as the highest welding pulse energy is 18 µJ. The outcome of unchanged absorption rate due to preheating to 200 °C under 10 ps and 184 fs is in agreement with the results of measured width of plasma-modified region *W_p_*, which shows independency on preheating to 150 °C. Contrary to *W_p_*, the width of heat affected zone *W_h_* depends on preheating; both 10 ps and 184 fs have enhanced *W_h_*; however, the 184 fs shows 13% higher *W_h_* compared to the 10 ps. Accordingly, the effects of preheating on glass welding under 10 ps and 184 fs conditions are mainly a mitigation of self-focusing discoloration, which only happens for the 184 fs pulse.

However, the wider *W_h_* does not contribute to enhancing welding strength in the current work, as welding mainly happens within the *W_p_*. While preheating to MT mitigates self-focusing discoloration and enhances *W_h_*, heat induced defects/microcracks could be the main reason behind lower shear strengths for glasses welded at MT compared to RT at 184 fs and 10 ps. Welding strength at RT using 184 fs in the “crack-free” region is higher by 42% compared to the 10 ps which could be due to the reduction in fracture toughness under the 10 ps pulses. Future work is required to investigate the preheating effects on glass material evolution under femtosecond and picosecond laser pulses in terms of fracture initiation and propagation.

## Figures and Tables

**Table 1:** Physical properties of glass slides [25].

Physical Property	Value
Density (g/cm^3^)	2.51
Refractive index	1.5
Heat capacity (J/kg.K)	2.2
Coefficient of thermal expansion (1/°C)	7.2 × 10^−6^
Melting point (°C)	827

**Table 2:** Laser transmission welding parameters of preheated glass slides.

	Pulse Energy (µJ)				
	4.5	6	10	14	18
Pulse Duration (fs) Hatch, *h* (µm)	184 200	184 200	184 200	X	X
Pulse Duration (ps) Hatch, *h* (µm)	X	10 200	10 200	10 300	10 400

## Data Availability

Relevant data obtained in this study are presented in this article.
